# Effectiveness of Palmitoylethanolamide (Levagen+) Compared to a Placebo for Reducing Pain, Duration, and Medication Use during Migraines in Otherwise Healthy Participants—A Double-Blind Randomised Controlled Study

**DOI:** 10.3390/ph17020145

**Published:** 2024-01-23

**Authors:** David Briskey, Rachael Skinner, Chelsie Smith, Amanda Rao

**Affiliations:** 1 RDC Clinical, Level 3, 252 St Pauls Terrace, Brisbane, QLD 4006, Australia; d.briskey@uq.edu.au (D.B.); rachael@rdcglobal.com.au (R.S.); chelsie@rdcglobal.com.au (C.S.); 2 School of Human Movement and Nutrition Sciences, The University of Queensland, Brisbane, QLD 4072, Australia

**Keywords:** PEA, migraine, pain, resolution, rescue medication

## Abstract

Migraines are a common neurological disorder that generally affects young to middle-aged adults and females more than males. Various treatment options are available; however, these can cause undesirable side effects. Therefore, alternative treatments with minimal side effects are still being investigated. Palmitoylethanolamide (PEA) is a signalling lipid known to have anti-inflammatory and analgesic properties. Previous prophylactic research has reported PEA supplementation to decrease pain associated with migraines. Upon commencement of migraine symptoms, participants were supplemented with either 600 mg of PEA (Levagen+) or a placebo (maltodextrin). Once a dose was taken, participants recorded a visual analogue scale (VAS) for pain every 30 min for 4 h or until the migraine resolved. If the migraine had not resolved 2 h post-dose, participants were instructed to take a second dose. Levagen+ supplementation resolved more headaches after 2- and 8 h, had a lower VAS for pain score at 1.5 and 4 h, and reduced rescue medication use significantly more than a placebo. No adverse events were reported in either group. Overall, PEA was safe and effective in reducing migraine pain, duration, and medication use in an otherwise healthy adult population.

## 1. Introduction

Migraines are one of the most common neurological disorders and a leading cause of disability worldwide [1,2]. Migraines are more prevalent in women [3] and younger to middle-aged adults [2], with ethnicity and lifestyle among other factors that contribute to the occurrence of migraines [2].

Migraine attacks are divided into distinct phases: the premonitory (prodrome), aura, headache, resolution, and postdrome phase [4]. The different phases can have variability in symptoms between sufferers and there can be an overlap of symptoms between each phase. The premonitory phase is generally associated with subtle symptoms up to two days before a migraine event. Symptoms may include neck pain, yawning, fatigue, mood changes, and sensory sensitivity [4].

Premonitory symptoms may last from a few hours to a few days [3]. The aura phase accompanies migraine attacks for about 20–30% of migraineurs. Aura can affect the visual field of both eyes and can last up to one hour. The headache phase is generally the most debilitating phase, which is the same for all who experience a migraine and can last for days. The most common symptoms of the headache phase are nausea, vomiting, and sensitivity to light, sound, and smell. The resolution phase (how a migraine is resolved) can be different for everyone. Resolution can include sleep, vomiting, medication, or simply time.

Due to the debilitating nature and variability in the aetiology of migraines, there is a wide range of treatment options available to assist with symptoms. Current treatment options include pharmaceuticals (e.g., triptans, paracetamol, ibuprofen, aspirin, beta-blockers, or blood pressure medication), lifestyle and behavioural modifications (e.g., avoiding triggers, diet changes, managing stress, hydration, and physical activity), and natural therapies (e.g., acupuncture and herbal supplements). Within the current treatment options available, there is a large cost variability, multiple routes of administration [5], and differing efficacy. Therefore, there is still a need for additional treatment options.

A potential new treatment option for migraines is palmitoylethanolamide (PEA). PEA is an endocannabinoid-like bioactive signalling lipid that is part of the N-acylethanolamine (NAE) family [6,7]. It is a highly lipophilic compound [8] that is synthesised in the lipid bilayer and found in multiple tissues within the body, including the brain [9]. It has been shown to be able to act as an anti-inflammatory and analgesic [7] and displays immunomodulatory and neuroprotective effects [8]. Its broad-spectrum analgesic and anti-inflammatory effects are due to its interaction and binding with peroxisome proliferator-activated receptor α (PPAR-α) and transient receptor vanilloid type 1 (TRPV1), as well as inhibiting mast cell activation resulting in the suppression of inflammation [10]. Together, the properties of PEA make it a feasible option to alleviate migraine symptoms.

Research conducted to date on PEA for migraine attacks has shown promising results from a range of different populations. A study by Papetti and colleagues supplemented 70 children (average age 10.3 years) suffering from migraines with 600 mg of ultra-micronized PEA (umPEA) for 3 months. Supplementation with umPEA decreased migraine frequency by greater than 50% per month in 63.9% of the study population [11]. The number of monthly attacks and pain intensity also decreased across the three-month trial period. In a study by Chirchiglia and colleagues in which 20 adults suffering from migraines with aura were supplemented with 1200 mg of umPEA daily for 90 days with nonsteroidal anti-inflammatory drugs (NSAIDs) used at first symptoms, supplementation with umPEA significantly reduced reported pain at day 60 and 90 [12] compared to a control group (NSAIDs only). Furthermore, there was a significant reduction in the number of attacks per month and the total days of pain experienced by individuals in the umPEA group, regardless of gender compared to a control group. A study by Hernandez and colleagues supplemented 25 adults suffering from episodic migraines with a combination of PEA (200 mg), Scutellaria, Boswellia, and Harpago every 12 h for 3 months. Supplementation with PEA significantly reduced the number of headache days per month, reduced the number of days of analgesic use per month, and reduced the reported pain severity [13].

Despite the reported positive outcomes of studies conducted to date on PEA in migraine sufferers, varying study designs mean an overall effect of PEA has been difficult to establish. Furthermore, no study has used PEA as a therapeutic treatment for migraine pain to date. Therefore, the aim of this study was to assess the effectiveness of PEA (Levagen+) on pain, duration, and conventional treatment (rescue medication) use during a migraine in an adult population. It was hypothesised that Levagen+ supplementation would reduce pain, duration, and rescue medication use during a migraine compared to a placebo.

## 2. Results

Of the 80 participants enrolled, 8 were lost to follow-up, 2 withdrew, and 6 completed the study but reported no migraine event. There was no difference in demographics between groups at the baseline (Table 1).

Of the 64 participants who recorded a migraine event, a total of 155 migraines were recorded (Table 2). There was no significant difference between groups for the number of migraines reported, the reported severity of migraine at onset, or the rescue medication use within the first 4 h (Table 2). There was a significant difference between groups for the number of migraines resolved at 2 h (Table 2).

Of the 61 participants who recorded a migraine event with no rescue medication use in the first 4 h, a total of 141 migraines were recorded. There was a significant difference between groups for the number of migraines resolved at 2 h. There was no significant difference between groups for the number of migraines reported or the reported severity of migraine at onset (Table 3).

Significantly more migraines that were classified as moderate at onset were resolved in the Levagen+ group compared to the placebo group (Table 4).

**Figure 1:**
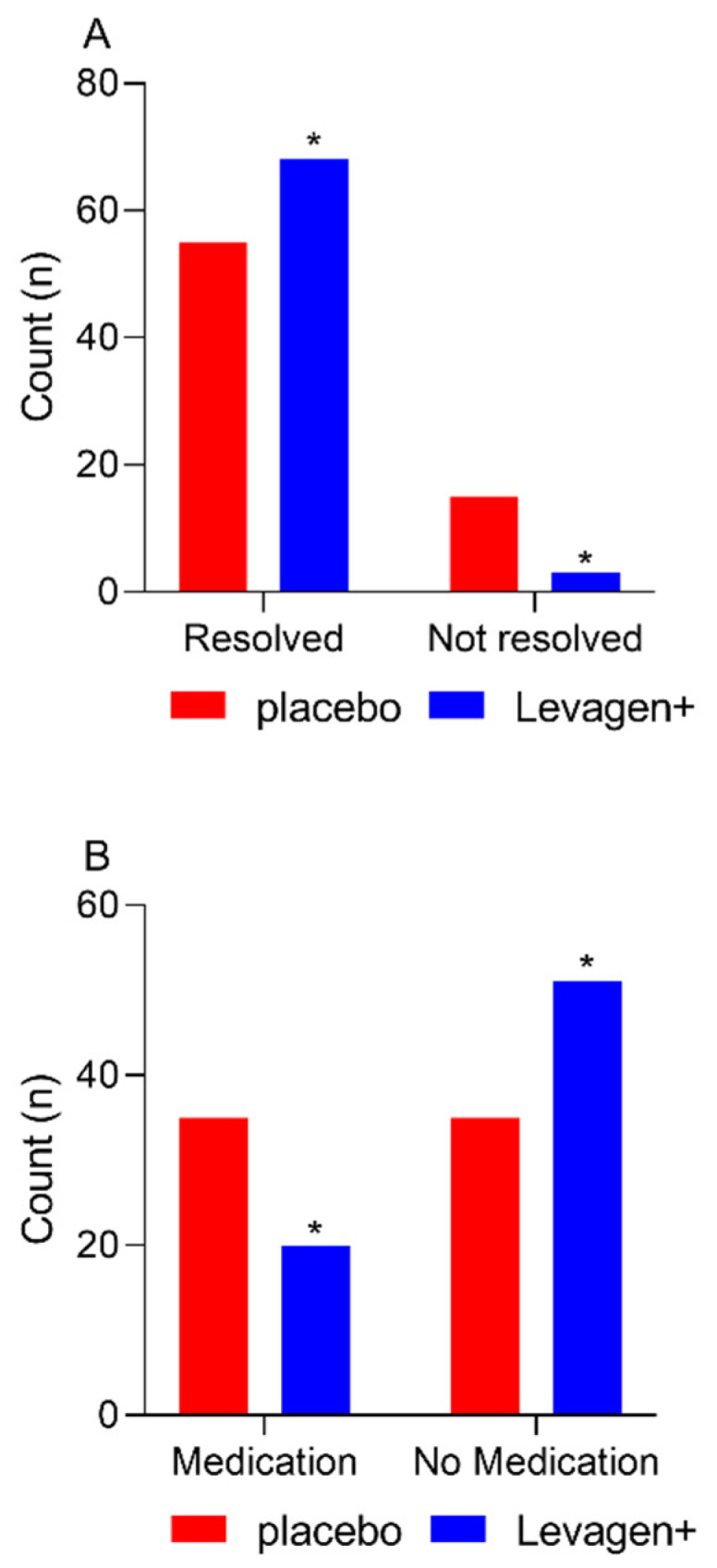
Migraine resolution and medication use for all participants reporting a migraine event and not using rescue medication within the first 4 h. (**A**) The number of migraine events resolved at 8 h. (**B**) The number of reported rescue medication use. * = significant difference to placebo group (*p* < 0.05).

At the completion of the 8 h, there were significantly more migraine events resolved in the Levagen+ group than in the placebo group (Figure 1A; 68 vs. 55; Chi-square *p* = 0.0022). There was no difference between groups for rescue medication use at each reporting period throughout the study (Table 5). There was significantly more rescue medication used overall during the study by the placebo group compared to the Levagen+ group (Figure 1B and Table 5).

**Figure 2:**
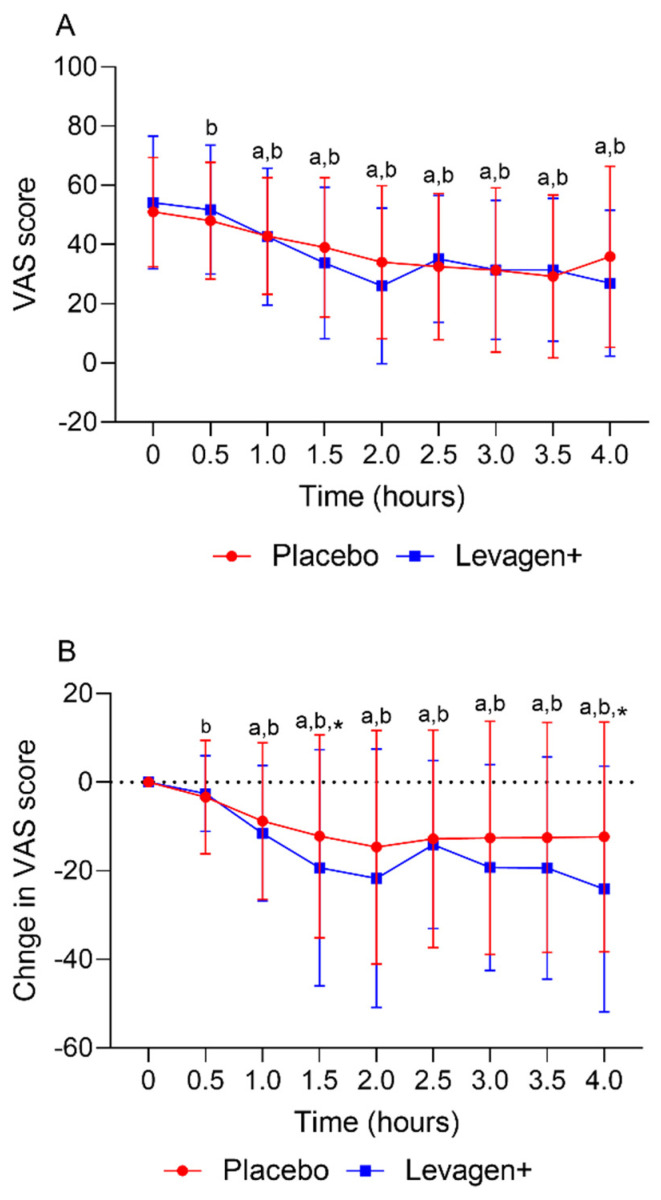
(**A**) Total VAS score over the first 4 h and (**B**) change in VAS score over the first 4 h. a = significant difference from the baseline in the Levagen+ group (*p* < 0.05); b = significant difference from the baseline in the placebo group (*p* < 0.05); * = significant difference between groups (*p* < 0.05).

The placebo and Levagen+ groups both reported a significant reduction in VAS scores (total and change from the baseline) from the baseline values from 30 and 60 min, respectively. There was no significant difference between groups at any time point for the VAS score (Figure 2A). The Levagen+ group reported significantly greater changes from the baseline in VAS scores at 1.5 and 4 h compared to the placebo group (Figure 2B).

## 3. Discussion

Palmitoylethanolamide has a long history in the scientific literature for its management of clinical conditions due to its reported analgesic and anti-inflammatory effects [14]. In the context of the current study on migraine pain, PEA is proposed to modulate pain by interacting with the endocannabinoid system and reducing inflammation [7,8]. When cells experience stress, PEA can be recruited to the site in response [8]. This protective role suggests that PEA, especially in large enough concentrations, may be suitable for alleviating pain and symptoms of migraines. PEA has also been shown to interact with nociceptor-specific ion channel TRPV1 and nuclear transcription factor PPAR-α [15]. These interactions reduce the transcription of pro-inflammatory genes to reduce inflammation and pain [16]. However, the evidence for the overall efficacy of PEA in treating migraines specifically needs to be determined.

This study aimed to assess the effectiveness of Levagen+ on pain, duration, and rescue medication use during a migraine in an adult population. Both groups were shown to be well matched, with no baseline differences between groups. Of the 80 participants enrolled, 64 reported at least one migraine. Participants reporting using rescue medication within the first 4 h were included in the whole group analysis (Table 2) but removed from the final efficacy outcome (Table 4). Of the remaining 61 participants, 141 migraine events were analysed for efficacy outcomes for the first 4 h. Over the first 4 h, both groups had a significant reduction in reported VAS scores compared to the baseline values. This effect could be due to a couple of possible reasons. One consideration is that migraine pain tends to dissipate gradually after a sudden onset. Therefore, it is expected that the VAS score for pain would gradually reduce over time. Another consideration is a potential placebo effect. External factors, such as a participant’s expectations from a clinical study, may play a role in pain relief. This highlights the importance of using a robust control group to differentiate the effects of a treatment from those of a placebo. Despite these possibilities, there was a significant difference between groups for the change from the baseline in VAS scores reported at the 1.5- and 4-h marks. This indicates a possible effect.

Supporting the change in VAS scores data, Levagen+ supplementation significantly resolved more migraines (i.e., pain-free) within 2 h compared to with the placebo group. This is a significant finding as migraine pain can persist for hours or even days, so fast relief is required. PEA was expected to alleviate migraine pain within the first 2 h due to its previously reported absorption profile. We have previously shown that Levagen+ appears in the blood within 30 min of consumption and peaks within 2 h of consumption [17]. When all migraine events were analysed up to 8 h, including any using rescue medication after 4 h, significantly more migraines resolved and significantly less rescue medication was used in the Levagen+ group compared to the placebo group.

The results of this study are in line with previous studies showing efficacy for PEA on migraine outcomes. A pilot study by Chirchiglia and colleagues supplemented people experiencing migraines with either 1200 mg of umPEA daily for 90 days and NSAIDs (ibuprofen, diclofenac sodium, or nimesuilde) used during acute migraine attacks (about 2 days for each attack) or only NSAIDs during acute migraine attacks. Over the 90-day treatment period, umPEA was able to significantly reduce pain in a time-dependent manner from day 60 to 90 compared to a control group (NSAIDs only) [12]. Supplementation with umPEA also resulted in a significant reduction in the number of days of pain and attacks per month, as well as a reduction in the use of NSAIDs. The results of the study by Chirchiglia are comparable to the results in the current study, showing a reduction in pain scores, migraine resolutions, and medication use. Chirchiglia and colleagues also reported no difference between genders. One of the observations of the current study is the greater number of female to male participants. However, as females are more prone to migraines than males, the proportion of males to females in the present study likely represents that of the general population that experiences migraines.

Despite the positive findings of the study by Chirchiglia, the design of the study makes it difficult to draw any conclusive evidence regarding the effect of PEA. Due to the presence of NSAIDs during the migraine event, it could be that PEA is aiding the NSAID effect, rather than having a direct physiological effect. The lack of a placebo in the control group also brings the study design into question. As the PEA group likely knew they were on the treatment, there was likely a strong placebo effect that was not accounted for. The number of participants is also a limitation. Although the study is classed as a pilot study, the seemingly low number of participants may reduce the power of the study. Future studies incorporating this design would benefit by having a placebo product supplemented daily along with the umPEA treatment and increasing the participant numbers.

A study by Hernandez and colleagues supplemented participants with Calmux^®^, containing 200 mg of PEA in combination with other natural products twice a day for 3 months. At the end of the treatment period, supplementation reduced the number of reported migraines, rescue medication use, and reported pain intensity [13]. These results are in line with those found in the current study, where there was increased efficacy in the moderate severity group for the Levagen+ treatment group compared to the placebo. The reduction in more severe pain scores may be due to the reported action of PEA being able to decrease the recruitment and activation of mast cells at pain sites, as well as inhibiting the activation of microglia [18]. Like the limitation in the study by Chirchiglia, there are several limitations to the study of Hernandez. The open-label design and setting in which it was conducted (a general practice office located in a hospital) make it possible for a strong placebo effect. The relatively low subject numbers also make it difficult to compare some of the outcome measures, like intensity.

Another consideration in conducting migraine-focused studies is the age of the population. With most migraine sufferers being the younger to middle-aged adult population, people of all ages can experience migraines [2]. While the population of the present study is representative of the general population that experiences migraines (i.e., 19–65 years old), PEA has also been shown to be effective in other populations. A study by Papetti and colleagues supplemented children experiencing migraines with 600 mg of PEA daily for 3 months. PEA supplementation was shown to decrease migraine frequency and intensity, resulting in a reduction in the number of reported severe attacks [11]. The results from the study by Papetti, combined with our results, demonstrate that PEA may benefit people of all ages who experience migraines.

One of the main differences between the present study and previous studies is that we supplemented as a treatment (at first onset), whereas previous studies have supplemented as a prophylactic (typically over 3 months). Despite this, both modes have proven to be beneficial in reducing migraine pain, frequency, and severity. Future studies may benefit from a combination of treatment and prophylaxis. By supplementing with PEA daily and again at the onset of migraine symptoms, it would ensure the maximum benefit of PEA for preventing or treating migraines.

Despite the positive outcomes of the present study, there are some considerations for any future studies. As mentioned, the timing of dose is something that would be worth considering. A combination of prophylactic and therapeutic dose may help prevent migraines from occurring in the first place. The dose of PEA is another consideration for future studies. The PEA dose used in the current study, 600 mg, is below the safe level of PEA consumption. Toxicology studies conducted in mice and rats have shown that the lethal dose for 50% mortality (LD50) is greater than the highest dose given of 2000 mg/kg of body weight. Furthermore, daily doses up to 1000 mg/kg body weight showed no toxicity effects [19]. The dose of PEA may potentially work in a dose-dependent manner, such that a stronger dose may have had greater efficacy again. Another consideration is the number of participants in the study. The current study is much larger than those conducted previously to date; more people may allow more differences between the PEA and placebo groups to be established.

Overall, this study, combined with previous studies, supports supplementation with Levagen+ being an alternative therapy for migraine pain. PEA was shown to decrease migraine pain and duration as well as reduce the use of rescue medication when compared with a placebo. PEA was found to be safe and tolerable amongst participants and in future, could be used more frequently as both a prophylactic and treatment for pain associated with migraines.

## 4. Methods

A randomised double-blind placebo-controlled study in healthy adults experiencing migraines was conducted. Participants were included in the study if they were 18 years or older; had no recent history (within 2 years) of clinically significant medical conditions, including but not limited to, malignancy (and treatment for malignancy), cardiovascular, neurological, psychiatric, renal, immunological, endocrine (including uncontrolled diabetes or thyroid disease), or haematological abnormalities that are uncontrolled; and had at least one migraine (not headache) episode every 2 months as classified according to the International Classification of Headache Disorders, 3rd Edition (ICHD3), for migraines published by the International Headache Society. A participant was considered to be suffering from a migraine when (I) at least five attacks fulfilled ICHD3 criteria B-D; (II) headache attacks lasted 4–72 h (untreated or unsuccessfully treated); (III) headaches had at least two of the four following characteristics: unilateral location, pulsating quality, moderate or severe pain intensity, and aggravated by or causing avoidance of routine physical activity (e.g., walking or climbing stairs); (IV) during headaches, at least one of the following occurred: nausea and/or vomiting, photophobia, and phonophobia; and (V) the migraine was not better accounted for by another ICHD3 diagnosis. Other inclusion criteria included the participant’s full agreement and ability to consent to participation in the study and access to a computer or smartphone for completing online questionnaires and events.

Participants were excluded from the study if they had chronic past and/or current alcohol use (>14 alcoholic drinks per week), used long-term medication (unless for controlled medical conditions as above), were pregnant, were trying to become pregnant, were lactating women, allergic, or hypersensitive to any of the ingredients in the active or placebo formula, used preventative medication, or reported migraines to occur on 15 or more days of the month for more than 3 months, which, on at least 8 days per month, had the features of a migraine headache, a debilitating attack lasting for more than 72 h, or a seizure.

Those who met the trial requirements were provided with detailed instructions on the study requirements prior to signing a consent form for enrolment in the trial. Once enrolled, participants had demographic data collected (age, weight, height, partner status, level of physical activity, alcohol and cigarette consumption, and work status) and were randomised to one of two groups: Levagen+ (active) or placebo. Randomisation was conducted by someone not involved in the study using a clinical study randomisation site (sealedenvelope.com) on a 1:1 ratio. The Levagen+ group received capsules containing 350 mg of Levagen+ (containing 300 mg of PEA and 50 mg of excipient) and the placebo groups received capsules containing 350 mg of a maltodextrin and microcrystalline cellulose mix per capsule. Both trial products appeared identical to ensure both the participant and investigator were blind. Each dose of the trial product consisted of two capsules, for a total of 700 mg of Levagen+ (containing 600 mg of PEA and 100 mg of excipient) or 700 mg of a maltodextrin and microcrystalline cellulose mix per dose. This dose was chosen as it has previously been shown to have efficacy [11,20].

Participants were instructed at the first onset of migraine symptoms to login to their participant-specific online trial diary and score their perceived migraine pain using a visual analogue scale (VAS) for pain. The system automatically logged the time and date of the entry. Following the baseline pain scoring of migraine symptoms, participants immediately supplemented their allocated product (two capsules of Levagen+ or placebo) with water. After supplementation, participants were instructed to score the migraine pain (using the VAS) every 30 min for the next 4 h or until the migraine was resolved. Participants were sent text messages every time a reporting point was due to ensure maximum data capture. A migraine was considered resolved and no further data entries were required when the participant scored a zero on the VAS for pain scale. If pain persisted for 2 h post migraine onset, participants were instructed to take an additional dose (two capsules) of their trial product but were still asked to refrain from any rescue medication use.

If pain did not subside within 4 h, rescue medication (e.g., ibuprofen or paracetamol) could be used. Any rescue medication used was recorded in the diary and the participant continued to score the migraine pain (VAS) every 60 min until the migraine was resolved or a further 4 h had passed since rescue medication was used (8 h from migraine onset). It was advised that, unless a migraine was experienced prior to the early afternoon, a participant should not take the supplement or record the event unless they knew they would be awake for at least 4 h. This was to prevent the participant entering their sleep period during the recording period. Within 24 h of dosing, participants recorded their gastrointestinal tract (GIT) tolerance via a questionnaire.

Participants were provided with enough product for 4 migraine episodes (16 capsules) and therefore could provide data for more than one migraine event. During the trial period, participants were followed up every 4 weeks until all four episodes occurred or their involvement in the study stopped (maximum 4 months).

The primary outcome measure was a reduction in migraine pain as assessed by VAS for pain. Secondary measures included a reduction in migraine duration, migraine severity, reduction in rescue medication use, gastrointestinal tolerability, and safety (adverse events).

Sample size was calculated using G*power (v3.0.10) accounting for an α error probability of 0.05 and powered to 0.95 for a 25% difference in change in pain scores (VAS) between groups. The resulting effect size was calculated as d = 0.8, with required group sizes of at least 35 events required. Therefore, allowing for dropouts, people reporting no event and people reporting multiple events, of group sizes of 40 participants, were chosen to allow an average of 1 event per participant (*n* = 80 participants total).

Analysis of efficacy (i.e., pain reduction) for the first 4 h was only conducted on data with no rescue medication use. If rescue medication was used within the first 4 h of data collection, that participant’s data were removed from analysis. A significant difference was considered at a level of *p* < 0.05. Data were analysed for both between and within-group differences. Migraine events were scored for intensity at onset based on the reported VAS score. VAS scores of 1–29 were classified as mild, 30–65 as moderate, and over 65 as severe.

Analysis was conducted using R (4.3.0) or GraphPad Prism (9.4.1), using a range of native statistical functions and, in some cases functions from the packages, tidyverse, dplyr, and ggplot. Slope analysis and some graphing was completed in Microsoft Excel (365). All results were first tested for normality before any other test was conducted. Based on the distribution of the data, the appropriate statistical test was used as required. Differences between groups were assessed using interdependent *t*-tests and covariates were accounted for with an ANCOVA. If a data point was missing, the previously reported score was used in the missing data point place.

## 5. Conclusions

This study was conducted according to the guidelines in the Declaration of Helsinki and all procedures involving human subjects were approved by Bellberry Limited, application number 2021-05-534. This trial was registered with the Australia and New Zealand Clinical Trial Registry (NCT05046522).

## Figures and Tables

**Table 1:** Baseline demographics for all trial participants.

	Levagen+ (*n* = 40)	Placebo (*n* = 40)
Age (years)	41.2 ± 11.5	43.8 ± 10.8
Age range (years)	26–60	19–65
Gender (M/F)	5/35	5/35
Height (cm)	165.5 ± 8.7	168.6 ± 7.3
Weight (kg)	69.5 ± 16.5	75.3 ± 17.0

Values are mean ± SD.

**Table 2:** All migraine events recorded.

	Levagen+ (*n* = 33)	Placebo (*n* = 31)
VAS at onset	55.0 ± 22.4	49.9 ± 18.5
Migraines (*n*)	76	79
Mild at onset (*n*)	9	14
Moderate at onset (*n*)	40	46
Severe at onset (*n*)	26	19
Resolved at 2 h (*n*)	27 *	14
Rescue medication use at 4 h (*n*)	5	9

Values are mean ± SD; * Significant difference to placebo.

**Table 3:** Final efficacy outcome for migraine events with no rescue medication used in the first 4 h.

	Levagen+ (*n* = 30)	Placebo (*n* = 31)
VAS at onset	54.2 ± 22.2	51.0 ± 18.3
Migraines (*n*)	71	70
Mild at onset	8 [11.3%]	11 [15.7%]
Moderate at onset	40 [56.3%]	43 [61.4%]
Severe at onset	23 [32.4%]	16 [22.9%]
Resolved at 2 h	27 *	14

Values are mean ± SD; * Significant difference to placebo.

**Table 4:** Proportion of resolved migraines based on severity at onset.

	Levagen+ (*n* = 71)	Placebo (*n* = 70)
Proportion pain free within 2 h		
Mild at onset (%)	11	10
Moderate at onset (%)	22 *	6
Severe at onset (%)	17	8
Proportion pain free within 4 h		
Mild at onset (%)	22	10
Moderate at onset (%)	39	28
Severe at onset (%)	36	27

* Indicates significant difference from placebo (*p* < 0.05).

**Table 5:** Occurrence of reporting rescue medication use after 4 h.

	Time (hours)					
	4	5	6	7	8	Overall
Placebo [*n* (total)]	9 (38)	16 (29)	5 (20)	3 (14)	1 (16)	35 (70)
Levagen+ [*n* (total)]	5 (31)	8 (21)	6 (14)	0 (7)	1 (4)	20 (71) *

*n* = number of reported rescue medication use; total = number of reported migraine events; * significant difference to placebo (*p* < 0.05).

## Data Availability

Data are available upon reasonable request from the corresponding author.
